# Non-linear measures of movement variability in multiple sclerosis: a clinical narrative review of Lyapunov exponent and entropy applications in balance and gait

**DOI:** 10.3389/fneur.2026.1839243

**Published:** 2026-05-26

**Authors:** Tina Banakheiri, Maya G. Panisset, Mary P. Galea, L. Eduardo Cofré Lizama

**Affiliations:** 1 Department of Medicine, Royal Melbourne Hospital, The University of Melbourne, Melbourne, VIC, Australia; 2 Department of Allied Health, School of Health Sciences, Swinburne University of Technology, Hawthorn, VIC, Australia

**Keywords:** disease progression, entropy, gait analysis, Lyapunov exponent, movement variability, multiple sclerosis (MS), nonlinear analysis, postural balance

## Abstract

Human movement variability, the natural fluctuation in motor performance across repeated tasks, is a fundamental characteristic of healthy biological systems, and its alteration is a hallmark of neurological dysfunction. The use of non-linear measures provides a powerful suite of complementary tools for capturing the complexity of this variability, revealing patterns in motor control that traditional linear metrics, based on time and distance, often miss. By quantifying aspects such as stability, adaptability, and the predictability of movement, these methods provide critical insights into neuromuscular function reflected in the dynamic variability of observed movements. This is especially valuable in multiple sclerosis (MS), where disruptions in sensorimotor pathways cause changes in movement patterns that can signal early dysfunction and may be able to guide targeted interventions. The purpose of this narrative review is to discuss the existing evidence for the clinical use of non-linear measures of walking and balance in detecting subtle changes, monitoring disease progression, and evaluating treatment effectiveness in MS.

## Introduction

### The hidden language of movement variability

Clinicians in neurorehabilitation may observe a range of atypical movement patterns in people with multiple sclerosis (MS). Some movements are uncontrolled, involuntary, and ataxic while others are stiff, over-controlled and cognitively demanding. Traditionally, this variability was attributed to meaningless “noise” in motor outputs, caused by neurological impairments affecting the motor control system (1). However, emerging evidence suggests that these fluctuations contain critical information regarding neural control mechanisms and may hold important prognostic value for neurorehabilitation (2).

Some degree of movement variability is healthy, reflecting the adaptability of neuromotor control in diverse environments (1, 3), whereas excessive variability can be pathological. Stergiou and Decker (2) presented the concept of optimal gait variability, a “sweet spot” between overly stable and overly chaotic patterns, which often correlate with pathological states. In MS, demyelination and lesions in cerebellar, sensory, or corticospinal pathways can disrupt this balance, leading either to excessive irregularity, or to abnormally consistent, inflexible movement patterns like those associated with spasticity (4). These contrasting manifestations highlight how the same disease process can produce distinct motor signatures depending on the specific neural systems affected. This raises an important question for clinicians: how can we better utilize and interpret these individual movement signatures to optimize rehabilitation outcomes?

**Figure 1:**
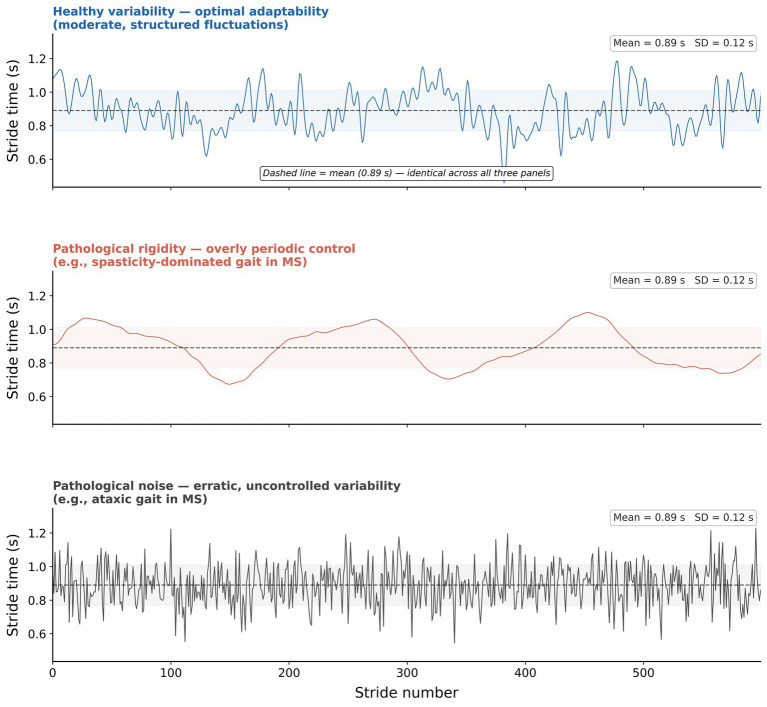
The limitation of averaging. Three different stride time patterns with identical means (0.89 s) and standard deviations (0.12 s), yet fundamentally different dynamic structures. Illustration created using AI-assisted graphic tools and refined by the authors.

Current measures of movement variability rely on spatiotemporal metrics such as range, standard deviation (SD), and coefficient of variation (CV), which do not capture important changes in movement patterns over time (5). They tell us how much movement fluctuates but cannot distinguish between, for example, adaptive flexibility and pathological instability. As shown in Figure 1, three fundamentally different stride time patterns can yield identical average values, masking critical information about motor control. Consider two patients with identical stride length variability: one exhibiting smooth, rhythmic variations, the other exhibiting erratic, unpredictable oscillations. Traditional assessments fail to differentiate these clinical cases (6, 7), as they are unable to quantify the performance of highly dynamical processes. This gap limits our ability to address key clinical concerns: Is observed movement variability positive, or negative? What rehabilitation strategies restore “healthy” variability?

## Variability and non-linear motor behavior

A system is non-linear when the effect of combined inputs is not the sum of their individual effects. This is true for the motor control system, which relies on physiological interactions of neurological, musculoskeletal, cardiovascular, respiratory, and metabolic systems (8). These systems interact across physiological time scales and adapt to different contexts, producing a coordinated motor behavior that is inherently variable in each repetition (9). This concept is illustrated by Bernstein’s study of professional blacksmiths (10). While their hammer strikes landed on target with consistency, the paths of their arms and joints varied with each swing. This “repetition without repetition” demonstrates that variability is not merely noise, but a functional feature of flexible, adaptive control. In MS, demyelination and disrupted sensory feedback interfere with systems interactions, resulting in unpredictable and less adaptable movements. Based on dynamical systems theory, non-linear metrics can provide clinically relevant gait assessments by revealing the hidden (dis-) organization of movement variability, even when changes may be subtle, thus overcoming limitations of traditional measures (11, 12).

Non-linear analysis techniques extract clinically meaningful information from raw movement time-series data, offering complementary insights into sensorimotor control by characterizing aspects of movement variability and temporal organization across multiple timescales. These non-linear metrics are derived from movement time-series signals, most commonly kinematic data such as trunk acceleration or segmental motion recorded during walking or standing tasks. Such signals can be obtained using laboratory-based systems such as optical motion capture or force plates, or wearable technologies such as inertial measurement units (IMUs), which record acceleration and angular velocity from specific body segments (13). The nature and location of the recorded signal, for example, trunk versus lower limb, or anteroposterior (AP) versus mediolateral (ML) direction, can influence the interpretation of these measures (14, 15). Throughout this review, the specific technology, sensor placement, and signal direction used in each study are reported alongside findings, with a comprehensive discussion of measurement considerations provided in a later section.

Beyond measurement considerations, these approaches show promise to provide three key capabilities: (1) detecting subtle neurological changes before clinical symptoms emerge (14, 16), (2) distinguishing between adaptive compensatory patterns and maladaptive pathological movements (2), and (3) quantifying rehabilitation improvements with high sensitivity (17). This enables clinicians to identify at-risk patients sooner, provide more personalized treatments, and more precisely track therapeutic outcomes. Although several non-linear metrics have been applied to gait and balance analysis, this review focuses specifically on the Lyapunov Exponent (LyE) and entropy measures, due to their clearer clinical interpretability and stronger evidence currently available in MS.

Despite strong validation in research, the translation of these powerful tools to clinical practice has been slow. As such, this review focuses on fundamental questions clinicians need answered:

- What do these non-linear measures reveal about sensorimotor neurological function?
- How can they be incorporated into the clinical decision-making workflow?
- What kind of technology is needed to implement these methods into practice?

This review addresses two major functional domains: balance control during standing and walking stability. These are of paramount clinical interest, as deterioration of mobility and balance are among the most common and disabling symptoms in people with MS (PwMS), regardless of disease trajectory (18). By examining how non-linear measures decode specific motor control deficits in each domain, this review aims to provide clinicians with a clear understanding of how MS disrupts movement organization and the potential implications for assessment and rehabilitation.

## Conceptual interpretation of non-linear measures in clinical practice

### The Lyapunov exponent (LyE): dynamic stability and response to perturbations

The LyE provides a clinically relevant measure of local dynamic stability (LDS), describing how the body responds to natural fluctuations during daily activities (19). The divergence rate, how quickly small movements deviate from a stable pattern, can be measured over different time ranges (e.g., 0 to 0.75 s during postural tasks) or cycle normalized ranges (e.g., 0 to 0.5 stride during walking, known as short-term divergence) (19). The latter is often reported as the local divergence exponent (LDE), which represents a finite-time estimate of LyE derived from kinematic, center of mass (CoM), or Center of Pressure (CoP) time series. LDE reflects the initial rate of trajectory divergence and is therefore particularly sensitive to the system’s ability to respond to small, naturally occurring perturbations during standing and walking (14).

These short-term windows capture the nervous system’s ability to correct small perturbations, making them the most clinically relevant for assessing fall risk. While linear measures quantify variability magnitude, LyE examines how natural movement variability changes over time, providing insight into how the nervous system maintains motor control (19). From a clinical perspective, LyE reflects how patients cope with minor perturbations during standing or walking, including weight shifts, distractions, and environmental changes (20).

**Figure 2:**
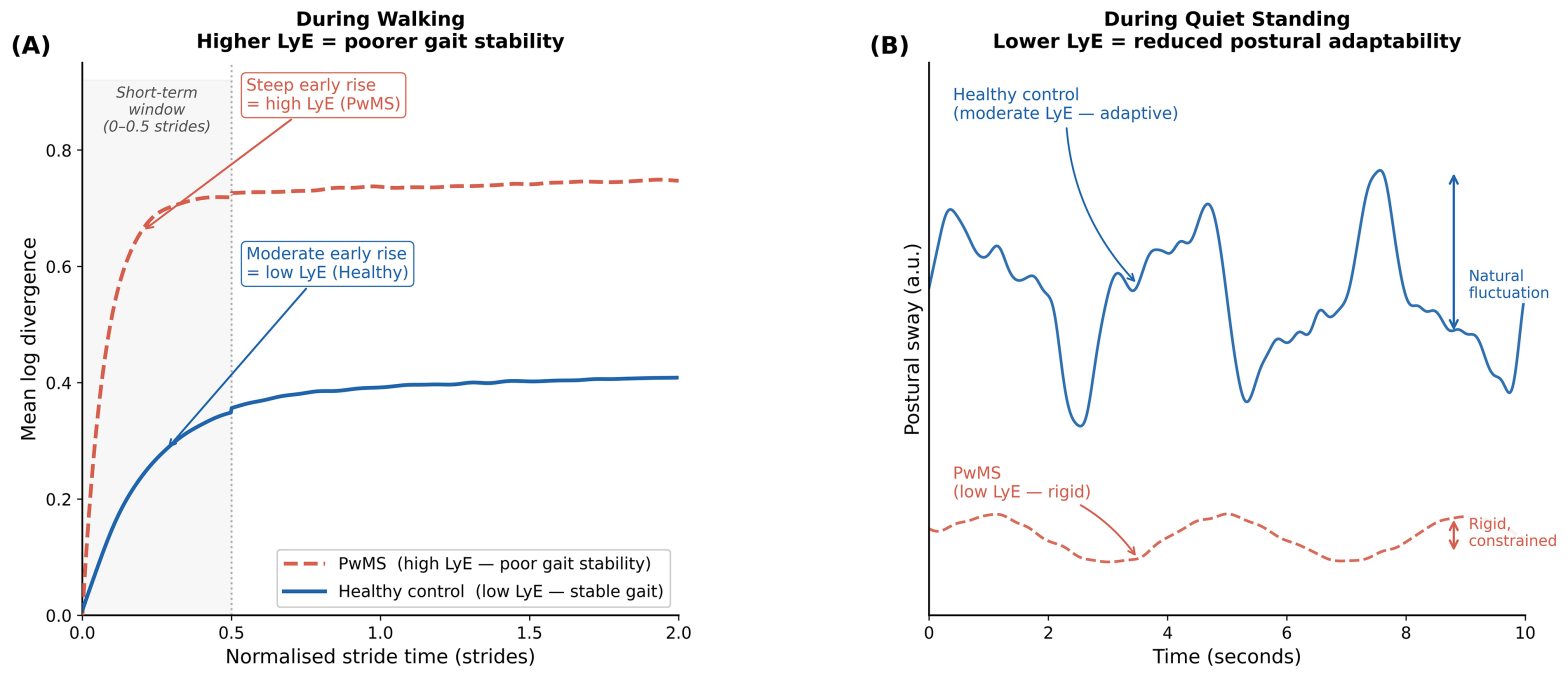
Task-dependent interpretation of the Lyapunov exponent (LyE). **(A)** Divergence curves during walking, where the slope of the mean log divergence within the short-term window (0–0.5 strides) reflects the LyE value, with a steeper early rise followed by a plateau indicating faster divergence and higher LyE in PwMS. **(B)** Postural sway signals during quiet standing illustrating the contrast between adaptive and rigid postural control in healthy controls and PwMS. Schematic representation for illustrative purposes.

Interpretation of LyE is task dependent, as illustrated in Figure 2. During walking, higher LyE values indicate worse stability, as small deviations rapidly diverge and are poorly controlled, reflected in patients whose gait seems unpredictable or who struggle to recover from minor disturbances. During standing, however, the opposite pattern can emerge: reduced LyE values in response to sensory challenges indicate a loss of adaptive capacity, where postural control becomes overly rigid and less able to respond flexibly. Low LyE during standing is therefore inherently ambiguous, it may reflect either genuine local stability or pathological rigidity. Interpretation is strengthened when considered alongside entropy-based complexity measures, which together provide complementary perspectives on the adaptive capacity of the postural control system. In healthy individuals, moderate LyE values reflect an optimal balance between stability and adaptability appropriate to the task demands (2).

LyE has particular value in MS rehabilitation, as it can detect declining stability early, even before clinical symptoms appear (14, 16). In postural control, LyE helps distinguish between healthy adaptive strategies and pathological instability (11, 21), while in gait analysis, it quantifies the effect of MS-related impairments on walking stability (22, 23).

### Entropy measures: regularity, predictability, and movement complexity

Entropy-based analyses provide insight into how movement patterns are organized over time by quantifying the regularity and predictability of motor output. Unlike spatiotemporal measures that quantify the amount of variability, entropy characterizes the *temporal structure* of movement, reflecting how likely specific patterns are to repeat from one moment to the next (24).

**Figure 3:**
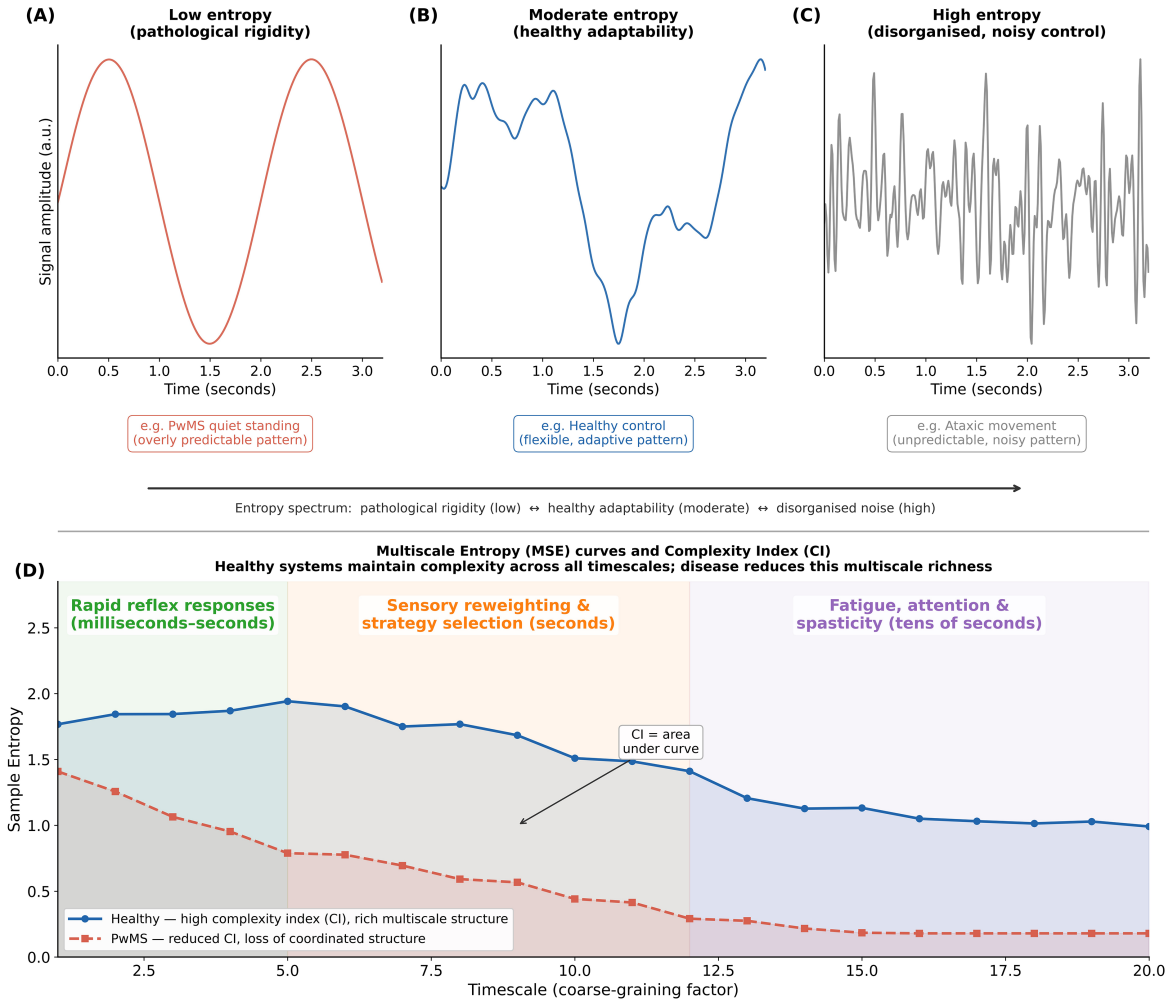
Entropy measures of movement complexity in people with multiple sclerosis. **(A–C)** Simulated signals illustrating the entropy spectrum from pathological rigidity (low entropy) through healthy adaptability (moderate entropy) to disorganized noise (high entropy). **(D)** Multiscale entropy (MSE) curves for healthy controls and PwMS. The shaded area under each curve represents the complexity index (CI). Schematic representation for illustrative purposes.

Single-scale entropy measures, such as Approximate Entropy (ApEn) and Sample Entropy (SampEn), quantify the regularity or predictability of a time series at a single temporal scale. Lower values indicate highly regular, repetitive patterns, which may reflect rigid or constrained motor control. Higher values suggest greater irregularity and lower predictability, potentially indicating disorganized or noisy patterns. Moderate, balanced values indicate the healthy adaptability essential for real-world functioning (25). Figure 3 illustrates this spectrum: from the highly regular, predictable signal characteristic of pathological rigidity, through the moderately variable pattern of healthy adaptability, to the disorganized noise reflecting impaired motor control. Part B of Figure 3 further illustrates how the Complexity Index summarizes multiscale entropy across physiological timescales, with healthy systems maintaining complexity across all scales, while disease reduces this richness.

ApEn is most effective for brief clinical assessments, reliably detecting early changes in movement regularity during short walking tests under 100 steps (26). SampEn addresses ApEn’s limitations for longer assessments by reducing measurement noise, making it preferable for tracking changes over time due to its greater consistency [for a comprehensive review, see (24)].

While single-scale entropy quantifies regularity, it does not capture the *structural richness* of movement organization across time scales. Multiscale Entropy (MSE) addresses this limitation by estimating sample entropy over multiple coarse-grained representations of the original signal, allowing assessment of movement complexity (27). In postural control and gait, these time scales reflect interacting mechanisms operating at different speeds: rapid reflex and muscle responses to perturbations (milliseconds–seconds), sensory reweighting and strategy selection such as ankle-hip transitions or stride-to-stride adjustments (seconds), and slower influences including fatigue, attention, and spasticity (tens of seconds to minutes) (28, 29). Complexity, in this context, refers to the presence of meaningful organized variability across all of these scales, a hallmark of healthy biological systems (29).

The Complexity Index (CI), derived from the area under the MSE curve, provides a summary measure of this multiscale structure. Higher CI reflects a movement system that exhibits rich, organized variability across scales, whereas reduced complexity suggests a loss of coordinated structure, a phenomenon commonly observed in aging and disease (29).

Crucially, the interpretation of entropy outcomes depends on the specific signal analyzed (e.g., sway, stride intervals, trunk acceleration), the task demands, and the comparison condition. Therefore, entropy measures are most informative when interpreted relative to healthy controls or task manipulations, rather than as absolute indicators of impairment (24). When interpreted alongside LyE, entropy measures help resolve the ambiguity inherent in divergence-based stability metrics alone, providing a more complete characterization of the postural control system.

## Balance control in people with multiple sclerosis

Balance control relies on the continuous integration of visual, somatosensory and vestibular inputs to maintain the body’s CoM within the base of support and respond adaptively to perturbations. In MS, inflammation and demyelination cause axonal damage and delayed neural conduction (4), disrupting the timing and reliability of sensory feedback and motor commands required for rapid postural adjustments (28). This impaired sensorimotor integration undermines stability even during quiet standing. Because the balance control system behaves as a non-linear dynamical system, where responses to perturbations are not proportional and vary with context, traditional linear measures of postural sway may fail to capture the full extent of these impairments (30). Therefore, non-linear analyses may offer additional insight into the temporal structure and adaptive capacity of postural control.

Non-linear analyses of CoP time-series recorded from force platforms during quiet standing have demonstrated that balance deficits in PwMS arise from a fundamental disruption in sensory reweighting, the process by which the nervous system dynamically adjusts reliance on available sensory inputs to maintain stability (30). In healthy individuals, removing visual input during eyes-closed standing triggers increased reliance on proprioceptive and vestibular information, preserving dynamic stability (21). PwMS exhibit the opposite response: LyE analyses have shown a significant reduction in sway divergence in both ML and AP directions under eyes-closed conditions, reflecting a more constrained and less adaptive postural output rather than improved stability (21). A significant group-by-visual-condition interaction confirmed this pattern, divergence decreased only in PwMS when vision was removed, indicating a failure to compensate through enhanced somatosensory or vestibular control (21). While lower LyE might superficially suggest greater stability, it instead reflects a reduced capacity to adapt to perturbations or changing environmental demands. This impaired reweighting likely reflects both slowed somatosensory conduction, evidenced by delayed spinal evoked potentials in PwMS (31), and increased reliance on previously successful but inflexible motor strategies (32). While sufficient for quiet standing, this rigidity becomes inadequate in dynamic or unpredictable environments, increasing fall risk.

Alongside LyE, Entropy measures provide a complementary perspective on postural adaptability, capturing the predictability of sway patterns rather than the rate of divergence. Roeing et al. (33) computed ApEn from CoP time series via a force platform during two 30-s eyes-open quiet standing trials in 30 PwMS with normal sway area and 36 healthy controls. Despite no group differences in any linear sway measure, including sway area, AP velocity and ML velocity, PwMS demonstrated significantly lower ApEn in the ML direction only, indicating greater time-dependent structure and reduced postural complexity. This excessive regularity reflects pathological rigidity in postural control, potentially arising from impaired supraspinal control increasing reliance on spinal reflex loops, delayed somatosensory conduction slowing corrective responses (31), and impaired sensory integration limiting adaptive capacity (34).

Motor control in the ML direction exhibits particular vulnerability in MS. Neuromuscular control of the pelvis in the frontal plane relies primarily on hip abductors and adductors, as neither the ankle nor knee joints allow substantial lateral adjustment during double-leg stance. AP balance, by contrast, can be managed across multiple joints with more fine-tuned stiffness modulation (35). MS exacerbates this ML vulnerability through asymmetric lower limb weakness and spasticity patterns that preferentially compromise lateral stability (36), explaining why patients who appear stable during standing may nonetheless struggle with dynamic real-world challenges (37).

For example, when sensory demands were increased, entropy measures revealed interesting task-dependent responses. During standing on foam with eyes closed, PwMS showed reduced SampEn in both ML and AP trunk acceleration directions compared to healthy controls, computed from a single IMU at the sternum in 81 early-stage PwMS and 38 healthy controls (38), indicating an inability to adapt postural strategies when proprioceptive input is degraded. However, under eyes-closed conditions on a firm surface, PwMS exhibited a paradoxical increase in SampEn in the AP direction, derived from IMUs placed simultaneously at the sternum and lumbar spine in 58 PwMS and 23 healthy controls, with the lumbar sensor demonstrating the strongest group differences (39). This pattern resembles anticipatory postural threat responses observed in healthy adults (40), suggesting maladaptive rather than functional variability. Together, this bimodal response, excessive rigidity on foam and maladaptive variability on firm surfaces, reflects an inability to scale postural strategies to environmental demands. Neurophysiological studies suggest this reflects corticospinal tract damage and increased muscle co-activation (41–43), disrupting movement automaticity, particularly during cognitive-postural interference (44).

MSE analyses extended these findings by evaluating postural complexity across multiple timescales. Busa et al. (45) computed the CI from CoP time series via force platforms during quiet standing and maximal lean conditions under normal and limited vision in 12 women with MS and 12 age-matched controls, finding consistently lower CI in PwMS in both AP and ML directions regardless of visual condition or postural challenge. Etzelmueller et al. (46) used a single force platform during 90-s eyes-open and eyes-closed standing trials in 54 PwMS divided into mild (EDSS 1–3, *n* = 31) and moderate (EDSS 4–6, *n* = 23) disability groups and 13 healthy controls. ML complexity deficits were most pronounced in moderately disabled individuals, with no significant differences emerging in mildly disabled individuals, suggesting that MSE in basic standing conditions may be insufficient to detect early complexity deficits (46). This was directly confirmed by Cofré Lizama et al. (11), who computed CI using MSE from IMUs placed simultaneously at the sternum and lumbar spine in 48 early-stage PwMS (EDSS ≤2.5) and 24 healthy controls across four standing tasks of increasing difficulty. Significant CI reductions in PwMS compared to controls emerged only during the most challenging condition, eyes closed on a compliant surface, at both sensor locations and in both planes, while simpler tasks revealed no group differences. These findings confirm that the balance control system must be sufficiently challenged to unmask subtle deterioration in sensorimotor integration in early-stage PwMS. This ML vulnerability is clinically significant, as ML sway measures are among the strongest objective indicators for differentiating fall risk in MS (47, 48). The influence of fatigue on postural complexity was examined by Santinelli et al. (49), who used a force platform during quiet bipedal stance in 13 minimally impaired PwMS (EDSS 0–2.5) and 12 healthy controls, finding that a calf-raise fatigue protocol reduced CI in the ML direction in both groups similarly with no between-group differences at baseline. This suggests that in early disease, fatigue may unmask latent postural instability through mechanisms shared with healthy individuals, implying that managing fatigue load may be sufficient to preserve postural stability in early-stage PwMS.

The clinical utility of these complementary measures were directly tested by Hunt et al. (50), who used LyE and ApEn values from quiet standing to classify 20 PwMS into two subgroups, those exhibiting chaotic instability characterized by high LyE, and those showing excessive rigidity characterized by low ApEn. A perturbation-informed intervention using balance-based torso-weighting shifted metrics in both subgroups toward a healthier intermediate range, indicating partial restoration of adaptable postural control, though the small sample size limits the generalizability of these findings. This is particularly meaningful because it demonstrates that two seemingly opposite presentations may share the same underlying deficit: an over-reliance on inflexible control strategies that fail under challenging or unpredictable conditions.

Taken together, LyE and entropy measures reveal that balance dysfunction in MS is not simply a problem of excessive sway, but a failure of flexible sensorimotor integration. PwMS exhibit impaired sensory reweighting, pathological rigidity under reduced sensory conditions, paradoxical maladaptive variability under other conditions, specific ML vulnerability that worsens with disability, and fatigue-induced unmasking of latent instability, none of which are captured by traditional sway measures. Crucially, the direction of the non-linear abnormality varies by sensory context and disability level, meaning that a single static assessment at rest likely systematically underestimates balance impairment in this population. These findings suggest that, in addition to using more sensitive measures, balance rehabilitation in PwMS should emphasize progressive sensory reweighting, reactive balance training to improve perturbation response time, and dual-task paradigms to enhance adaptability under realistic cognitive-motor demands, with careful attention to fatigue load, particularly in early-stage patients where fatigue may be the primary factor unmasking latent postural instability.

## Dynamic gait stability in multiple sclerosis

Walking is central to independence and quality of life, and mobility deficits in neurological disorders are linked to higher rates of morbidity and mortality (51). In MS, walking difficulties can emerge early, even before clinical signs of pyramidal dysfunction (52), stemming from impairments across sensorimotor, visual, and cognitive systems that lead to weakness, spasticity, impaired balance and coordination (53). These deficits contribute to fall rates of approximately 50% in PwMS (54), exceeding those observed in older adults (55).

Non-linear measures have revealed that this gait dysfunction reflects something deeper than reduced speed or step length. It represents a failure of the sensorimotor system to manage the natural perturbations that occur with every stride. Caronni et al. (22) recorded trunk acceleration via a single IMU at the lower back (L5) during a Six-Minute Walk Test (6MWT) at maximum speed in 80 minimally impaired PwMS (EDSS ≤2.5) and 20 healthy controls. PwMS walked significantly slower than controls and demonstrated significantly larger short-term LyE (sLyE), with group differences remaining significant even after statistically accounting for this speed difference. sLyE measures how quickly small step-to-step errors grow over 0 to 0.5 strides (25), detecting subtle instability not apparent in routine assessments. Crucially, this finding indicates that gait instability in early MS is not simply a consequence of walking more slowly, but the sensorimotor system itself is less able to recover from small perturbations regardless of pace. Directional analysis from the same study revealed that AP instability at the lower trunk (L5) was the most clinically informative direction, correlating significantly with reduced walking speed, impaired balance on the Fullerton Advanced Balance scale and Timed Up and Go test, and greater disease impact including fatigue and self-reported walking limitations. By contrast, sLyE at the lower trunk in the ML and vertical directions did not correlate with any clinical measures. Extending this to upper trunk assessment, Cofré Lizama et al. (14) found that when sensors were placed simultaneously at the sternum and lumbar spine during overground walking in 49 minimally impaired PwMS (EDSS ≤2.5), sternum-derived LDE, particularly in the ML and AP directions, provided superior classification accuracy compared to lumbar alone. This suggests that upper trunk instability in the ML direction may reflect a particularly sensitive early marker of MS-related gait impairment, potentially representing compensatory movement of the upper trunk in response to the AP instability detected at the lower trunk level. In healthy walking, spinal central pattern generators operate with minimal supraspinal involvement (56), enabling automatic perturbation correction. In PwMS, demyelination along ascending and descending pathways (57) disrupts this coordination by delaying sensory feedback (58) and supraspinal transmission (59), likely explaining why PwMS adopt slower speeds as a compensatory strategy.

The clinical consequences of this instability are substantial. Tajali et al. (23) assessed trunk kinematics via a 7-camera motion capture system, with a marker cluster placed at the level of T7, during treadmill walking in 70 PwMS (EDSS 0–5.5) and followed participants prospectively for 6 months. Short-term LyE computed from three-dimensional trunk kinematics under single-task walking was the only significant independent fall predictor, with 43% of participants experiencing at least one fall. Importantly, this predictive capacity remained significant after accounting for disability level, demonstrating sLyE’s value as an independent fall risk tool beyond what clinical scales alone can provide.

Beyond rested baseline assessment, fatigue substantially exacerbates gait instability and must be considered in any comprehensive fall risk evaluation. Leone et al. (60) documented walking-related motor fatigue, defined as a decline of 15% or more in distance walked between minute 1 and minute 6 of the 6MWT, in 208 PwMS (EDSS up to 6.5). Fatigue affected approximately one-third of PwMS overall, rising to nearly half of those with moderate-to-severe disability (EDSS ≥4.5). Arpan et al. (61) directly linked this performance decline to dynamic stability, computing LyE from IMUs at the sternum and lumbar spine during the 6MWT in 25 PwMS (median EDSS 3.5) and 10 healthy controls. No stability differences were found between groups during the first 3 mins of walking. Differences only emerged from minute 4 onwards, with approximately 36% of PwMS showing a significant decline in LDS over the course of the 6MWT. The decline in stability correlated significantly with the decline in walking distance, confirming that fatigue directly compromises the sensorimotor resources needed for safe locomotion. McLoughlin et al. (62) provided further evidence, finding that a 6MWT induced significant increases in postural sway and reductions in lower limb strength in 34 PwMS with moderate disability (mean EDSS 3.7) but not in 10 healthy controls, with sway increases under eyes-closed conditions correlating with both acute and self-reported fatigue. Together, these findings suggest that fatigue-induced deterioration may broadly reflect neuromuscular system collapse and support the performance of stability assessments both before and after fatigue-inducing activities to fully capture fall risk.

The trunk acceleration pattern during walking reveals additional mechanistic detail. Huisinga et al. (63) computed LyE simultaneously from IMUs at the sternum and lumbar spine (L5) during 30 s of steady-state overground walking in 15 PwMS (mean EDSS 4.21) and 15 speed-matched healthy controls, finding greater divergence in both ML and AP directions in PwMS across both sensor locations. Craig et al. (64) found that all groups showed disproportionate trunk destabilization when somatosensory input was reduced via foam-soled shoes, but only MS fallers showed additional sagittal plane (AP) destabilization when vision was altered, a pattern absent in non-fallers and healthy controls. This indicates that fall-prone PwMS are specifically reliant on visual feedback to regulate trunk-foot coordination during walking.

From a neuroanatomical perspective, these stability impairments have a measurable structural basis. Cofré Lizama et al. (16) assessed LDE from motion capture markers at the sacrum, shoulder and cervical spine during treadmill walking in 25 minimally impaired PwMS. Moderate but significant correlations were found across all marker locations between poorer gait stability and reduced fiber density in the corticospinal tract and with interhemispheric sensorimotor tract damage at the cervical level. These findings position LDE as a functional, cost-effective biomarker of white matter degeneration detectable before clinical disability becomes evident. In a longitudinal study, Gervasoni et al. (65) computed sLyE from a lower back IMU (L5) during the 6MWT at two time points 2 years apart in 56 early-stage PwMS, finding no group-level changes in EDSS, 6MWT distance or self-reported walking ability, yet AP gait instability correlated significantly with EDSS change across the whole sample. In the 30% who deteriorated clinically, ML gait instability, stride regularity (assessed via autocorrelation of trunk acceleration) and ML symmetry together explained 56% of variance in EDSS change, and AP instability correlated with patients’ perception of worsened balance during daily activities. Together these findings demonstrate that instrumented stability measures can capture meaningful disease progression that remains invisible to standard clinical tools. While longitudinal evidence remains limited, these findings provide preliminary support for the potential of non-linear gait measures as sensitive markers of disease progression in MS, warranting larger prospective studies to establish their responsiveness and predictive power over clinically meaningful timeframes.

Complementing this evidence, LyE has also demonstrated responsiveness to rehabilitation-induced change in dynamic gait stability. Hilfiker et al. (17) computed LDS using the largest Lyapunov exponent from a triaxial accelerometer at the lumbar spine (L3) during a 3-min walking test in 18 PwMS (EDSS 3–6.5) undergoing a three-week inpatient rehabilitation program. They found that LDS improved across all trunk acceleration directions, with the greatest effect in the AP direction, followed by ML and vertical directions. Critically, these stability improvements showed larger effect sizes than those observed in commonly used clinical gait measures including the 10-meter walk test and 3-min walk distance (17). The rehabilitation-induced stability gains occurred alongside reductions in pain, fatigue, and spasticity and improvements in static balance and wellbeing, suggesting that LDS captures multifactorial therapeutic recovery that speed-based tests cannot. These findings suggest that LDS shows promise as an outcome measure for evaluating treatment effectiveness in MS, capturing therapeutic gains in gait quality that conventional measures of speed and walking distance may underestimate. However, evidence remains limited and larger trials are needed to confirm these findings.

Entropy analyses provide complementary insight into gait organization and adaptability in PwMS. Kaipust et al. (66) computed ApEn from stride length and step width derived from 3D motion capture during treadmill walking in 10 PwMS (mean EDSS 3.95) and 10 healthy controls. Greater stride length variability, measured by CV, only emerged in more severely impaired individuals (EDSS ≥4), suggesting linear metrics may only detect gait alterations at advanced stages of disease. In contrast, they found significantly lower ApEn in PwMS overall, despite similar amounts of variability on linear measures, highlighting entropy’s superior sensitivity for detecting the structural degradation of gait patterns that magnitude-based measures miss entirely. The authors attributed this pathological regularity to widespread demyelination reducing the degrees of freedom available to the locomotor system, increasing reliance on rhythmic spinal pattern generator activity at the cost of flexible, adaptive stepping. Shema-Shiratzky et al. (67) demonstrated that this rigidity is further eroded by fatigue during sustained walking, instrumenting the 6MWT with IMUs at the lower back and ankles in 58 PwMS across mild and moderate disability levels. Cadence, stride time variability, stride regularity and SampEn all significantly deteriorated across the test while gait speed did not change. A critical group-by-fatigue interaction emerged: PwMS with mild disability maintained stable rhythm and complexity throughout the entire 6 min, whereas those with moderate disability showed significant declines from the second minute onwards. Fatigue burden and self-reported gait disability correlated strongly with fatigability across regularity and variability domains, while gait speed changes showed no association with fall history, underscoring that velocity is an inadequate surrogate for the adaptive control deficits that drive fall risk. These patterns allow clinicians to objectively measure what they often observe: patients who walk adequately at the start of a task but become progressively unstable as activity continues, particularly those with moderate disability.

Taken together, entropy and LyE measures reveal that gait dysfunction in MS is not simply a reduction in speed or step regularity, but a fundamental failure of adaptive locomotor control. PwMS demonstrate more predictable and constrained gait patterns, reduced ability to cope with step-to-step perturbations, directional vulnerability particularly in the AP direction at the lower trunk and ML direction at the upper trunk, and progressive destabilization under fatigue, all of which carry stronger associations with fall risk and patient-reported disability than velocity alone. Fall-prone PwMS also show specific impairment in sensory reweighting during walking, becoming disproportionately reliant on visual feedback when somatosensory input is reduced. These findings reflect a shift toward rigid, simplified control strategies driven by corticospinal and interhemispheric tract degeneration, compounded by impaired sensory reweighting. Clinically, gait rehabilitation should target adaptability and dynamic stability rather than distance or speed, incorporating lateral weight-shifting, hip abductor strengthening, dual-task walking and graded sensory challenges. Given that fatigue accelerates gait deterioration from as early as the second minute of sustained walking in moderately disabled PwMS, rehabilitation programs must adopt a fatigue-aware approach that regulates duration, task difficulty and rest periods, not to avoid challenge, but to prevent the reinforcement of maladaptive simplification that emerges when patients train beyond their sensorimotor capacity.

## Beyond the naked eye: quantitative tools for movement analysis

Measurements of movement complexity and stability have evolved through technological advances. These analyses were initially limited to laboratory settings, using equipment such as optical motion capture systems and force plates. The data they provide are accurate and remain the gold standard. However, their clinical utility is restricted by high cost, specialized expertise, and constrained environments that may not elicit natural movement patterns (13).

### The wearable revolution: from lab to clinic and home

**Figure 4:**
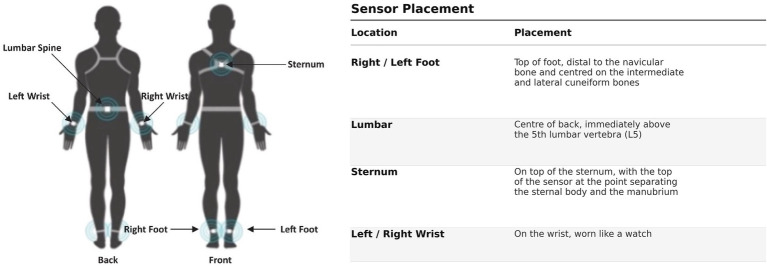
Recommended sensor placements for clinical analysis [adapted from Ref. (80), CC BY 4.0].

Wearable inertial measurement units bridge the gap between laboratory precision and clinical practicability (13, 68). Containing accelerometers and gyroscopes, they can be attached to specific anatomical locations (Figure 4), enabling assessments in clinics or at home.

For LyE analysis, optimal placements include the lower back (lumbar spine near L5 vertebra), which measures whole-body CoM movement, and the sternum (upper trunk) to evaluate trunk control and upper body stability (14, 15). Sternum placement has demonstrated superior classification accuracy for identifying PwMS, even in the earliest stages without evident gait impairments (69). Additionally, sternum-derived stability measures have shown significant correlations with clinical impairment, fatigue and self-reported walking limitations (22, 69). The choice between lumbar and sternum placement may reflect different aspects of motor control; while the lumbar sensor is a proxy for CoM motion, increased instability at the sternum may indicate that the balance control system prioritizes CoM stability, with the upper trunk exhibiting compensatory movements as a consequence of central nervous system damage (15, 69). While this relationship is yet to be fully elucidated, future work to develop and validate a clinical mechanistic understanding of these complementary non-linear measures could advance work, e.g., in computational modeling (70) or clinical reasoning frameworks (71), toward precision neurorehabilitation.

Across the studies reviewed, the number of sensors used varied considerably, from a single force platform or IMU to multi-sensor arrays, and this variability has direct implications for clinical implementation (Table 1). For standing balance assessment, a single force platform remains the gold standard for CoP-derived entropy and LyE measures (29, 33, 46, 49). When wearable sensors are used instead, a single IMU at the sternum emerges as the preferred alternative: direct comparison of sternum and lumbar placements during an instrumented standing balance task found that the sternum produced effect sizes approximately 39% larger than the lumbar sensor for distinguishing early-stage PwMS from healthy controls (38). For walking stability, a single lumbar IMU at L5 has demonstrated sufficient sensitivity for detecting sLyE differences in minimally impaired PwMS, monitoring longitudinal disease progression over 2 years, and capturing rehabilitation-induced change with effect sizes exceeding those of conventional clinical measures (17, 22, 65). When the primary clinical goal is early detection and classification of PwMS during walking, a single sternum IMU provides superior classification accuracy compared to walking speed alone (14). Critically, adding a lumbar sensor alongside the sternum produced only marginal additional gain in classification accuracy (14), suggesting that a single sternum-mounted IMU represents a practical and clinically efficient minimum for walking-based non-linear assessment in early-stage MS. A two-sensor approach combining sternum and lumbar may nonetheless be preferable when both classification accuracy and directional specificity, for example, distinguishing AP from ML instability, are simultaneously required. When assessing whole-body coordination during walking under challenging sensory conditions, a trunk-ankle sensor pair provides unique information about upper-lower body relationships that a single trunk sensor cannot capture (64). Beyond two sensors, the available evidence does not support meaningful gains in non-linear metric quality from additional IMU placements. The largest multi-sensor study in this review computed LyE from sternum and lumbar sensors only, using additional sensors exclusively for secondary purposes such as turn detection and distance measurement (61).

**Table 1:** Sensor configurations used across reviewed studies for non-linear gait and balance assessment in people with multiple sclerosis.

Study	Task	Sensor type	No. of sensors	Placement	Axes	Metric	Key sensor finding
Roeing et al. (33)	Quiet standing, eyes open, 30s	Force platform	1	-	AP, ML	ApEn	Single platform detected ML ApEn deficit despite normal linear sway measures
Busa et al. (45)	Quiet standing + maximal leans, eyes open/closed	Force platform	1	-	AP, ML	MSE/CI	Single platform detected CI differences across all postural conditions
Etzelmueller et al. (46)	Quiet standing, eyes open/closed, 90s	Force platform	1	-	AP, ML	MSE/CI	Single platform sufficient; ML CI deficits most pronounced in moderate disability
Santinelli et al. (49)	Quiet bipedal stance, pre/post fatigue	Force platform	1	-	AP, ML	MSE/CI	Single platform detected fatigue-induced CI reduction in both groups
Carpinella et al. (38)	Modified Romberg, foam, eyes closed, 30s	IMU	1	Sternum	AP, ML	SampEn	Sternum produced ~39% larger effect sizes than lumbar for standing balance in early-stage PwMS
Cofré Lizama et al. (11)	Quiet standing, 4 sensory conditions, 60s	IMU	2	Sternum + lumbar	AP, ML	SampEn	Lumbar AP showed strongest group differences under eyes-closed hard surface condition
Caronni et al. (22)	6MWT, maximum speed	IMU	1	Lower back (L5)	AP, ML, VT	sLyE	Single L5 sensor detected sLyE differences in EDSS ≤2.5 after controlling for walking speed
Cofré Lizama et al. (14)	5-min overground walk	IMU	2	Sternum + lumbar	AP, ML, VT, 3D	LDE	Sternum alone nearly matched combined sternum+lumbar classification accuracy
Cofré Lizama et al. (16)	5-min treadmill walk, fixed speed	Motion capture	3 marker locations	Sacrum, shoulder, cervical (C7)	3D	LDE	All three locations detected group differences; sacrum LDE most strongly associated with CST damage
Tajali et al. (23)	2-min treadmill walk, preferred speed	Motion capture	1 cluster	T7 trunk	3D	LyE	Single trunk marker cluster sufficient to predict prospective falls over 6 months
Huisinga et al. (63)	30s overground walk, self-selected speed	IMU	2	Sternum + lumbar (L5)	AP, ML	LyE	Both locations detected group differences; no significant group-by-sensor position interaction
Craig et al. (64)	Treadmill walk, 3 sensory conditions, 90s	IMU	2	Lumbar (L4-L5) + ankle	Frontal, sagittal	LyE, SampEn	Trunk-ankle pair uniquely captures whole-body coordination unavailable from single trunk sensor
Arpan et al. (61)	6MWT, maximum speed	IMU	6	Lower back, sternum, feet, wrists	AP, ML	LyE	LyE computed from sternum and lumbar only; additional sensors used for turn detection and distance measurement
Gervasoni et al. (65)	6MWT, 2-year follow-up	IMU	1	Lower back (L5)	AP, ML	sLyE	Single L5 sensor sufficient for longitudinal disease progression monitoring over 2 years
Hilfiker et al. (17)	3-min walk, maximum safe speed	Accelerometer	1	Lumbar (L3)	AP, ML, VT	LDS (largest LyE)	Single lumbar sensor detected rehabilitation-induced improvements exceeding conventional measures
Kaipust et al. (66)	3-min treadmill walk, self-selected speed	Motion capture	Multiple	Bilateral lower extremity	Stride length, step width	ApEn	Stride-derived ApEn detected regularity differences undetected by linear measures
Shema-Shiratzky et al. (67)	6MWT, self-selected pace	IMU	2	Lower back + ankles	AP, ML	SampEn	Lower back primary for SampEn; ankle sensors used for spatiotemporal parameters only

IMU, inertial measurement unit; CoP, center of pressure; AP, anteroposterior; ML, mediolateral; VT, vertical; ApEn, approximate entropy; SampEn, sample entropy; MSE, multiscale entropy; CI, complexity index; sLyE, short-term Lyapunov exponent; LyE, Lyapunov exponent; LDE, local divergence exponent; LDS, local dynamic stability; 6MWT, six-minute walk test; PwMS, people with multiple sclerosis; EDSS, Expanded Disability Status Scale; CST, corticospinal tract. Force platform studies have no sensor placement as CoP is derived from the platform itself.

### Smartphones and remote monitoring

Building on wearable IMUs, embedded sensors in smartphones have been validated to assess gait parameters such as cadence, step count, and turning speed, showing strong agreement with clinical tests and research-grade sensors in neurological conditions (72–74). Critically, recent work has extended this to non-linear stability metrics. Bastani et al. (75) computed LDE from a smartphone placed on the sternum during a 6MWT in 20 older and 20 young adults, finding excellent agreement with a research-grade wearable IMU (ICC > 0.75 across all movement directions). Direction-specific analyses revealed higher ML instability in older adults, consistent with the ML vulnerability identified in PwMS throughout this review, suggesting that directional LDE from smartphones may capture clinically meaningful stability differences. Smartphones facilitate frequent, unsupervised assessments in natural environments, capturing performance often overlooked in labs or clinics (74). This is important for PwMS in remote areas or with limited mobility, who demonstrated greater uptake of mobility-supportive telehealth during and after the COVID-19 pandemic (76), as smartphones can objectively track progression and provide data to clinicians remotely.

A review by Strongman et al. (77) on smartphone accelerometers for gait analysis concluded that smartphones could provide accurate, reliable, and valid kinematic data when compared with motion capture and pressure walkways. However, practical considerations remain: body placement (e.g., trouser pocket, hand, chest, or belt bag) affects consistency, most validation studies were conducted in controlled settings, and LDE estimates are currently device-specific, findings from one phone model may not directly translate to another without revalidation (75).

### From group findings to individual interpretation: Are we there yet?

One of the ongoing challenges in applying nonlinear gait metrics in a clinical setting is the absence of reference thresholds. It is scientifically interesting to discover differences between groups of PwMS and healthy controls, providing early validation. However, clinicians need to determine whether a specific patient’s value is normal. Without cut-off values, even the most sensitive measure remains mostly a research tool (74, 78).

Work has recently begun to bridge this gap. Carpinella et al. (78) used inertial sensors during 6MWT to establish cut-off values for several gait measures in PwMS, including the sLyE for AP dynamic instability. They found that more than half of individuals with mild MS, even without visible gait problems, already showed abnormal stability values. Similar relationships were found for ML gait symmetry. Importantly, these findings were confirmed in a separate group, indicating that the limitations were not specific to just one sample. The percentage of abnormal values also increased with higher disability and fall risk, suggesting that these calculations reflect significant clinical decline (78). This is an important step: non-linear measures are moving toward individual-level interpretation rather than being limited to group comparisons.

However, cut-off values alone are insufficient. Identifying a value outside a reference range does not clarify its implications for treatment decisions nor its potential to change following rehabilitation. A systematic review by Abou et al. (74) confirmed that smartphone-based gait measures in MS are generally valid and reliable, but responsiveness over time, minimal detectable change, and minimal clinically important difference are largely unknown. In practice, we cannot yet determine whether changes in LyE after therapy reflect meaningful improvement or measurement variability (74).

A framework by Karatsidis et al. (79) attempted to standardize digital gait assessment by organizing metrics into clinically meaningful domains such as speed, rhythm, stability, symmetry, variability, smoothness, complexity, and fatigue. Nonlinear measures, including LyE and entropy, are situated within stability and complexity, providing a common language. However, interpretation remains incomplete, and we do not yet know how to contextualize complexity values for age, disability level, or task demands, nor is there consensus on optimal calculation parameters (24, 79).

## Conclusion

Non-linear metrics are best viewed not as standalone clinical tests, but as complementary tools that deepen our understanding of motor control alterations in MS. By quantifying subtle changes in walking and balance dynamics, these measures may contribute to more individualized rehabilitation planning as the evidence base continues to grow. Understanding non-linear measures of walking and balance enables clinicians to read emerging literature with greater precision and identify opportunities to tailor rehab interventions more precisely.

## References

[1] Davids K Bennett S Newell KM Movement system Variability: Human Kinetics. Champaign (2006)

[2] Stergiou N Decker LM. Human movement variability, nonlinear dynamics, and pathology: is there a connection? Hum Mov Sci. (2011) 30:869–88. doi: 10.1016/j.humov.2011.06.002, 21802756 PMC3183280

[3] Dingwell JB Cusumano JP. Nonlinear time series analysis of normal and pathological human walking. Chaos Interdiscip J Nonlinear Sci. (2000) 10:848–63. 10.1063/1.1324008 12779434

[4] Bjartmar C Trapp BD. Axonal and neuronal degeneration in multiple sclerosis: mechanisms and functional consequences. Curr Opin Neurol. (2001) 14:271–8. doi: 10.1097/00019052-200106000-00003, 11371748

[5] Harbourne RT Stergiou N. Movement variability and the use of nonlinear tools: principles to guide physical therapist practice. Phys Ther. (2009) 89:267–82. doi: 10.2522/ptj.20080130, 19168711 PMC2652347

[6] Palmieri RM Ingersoll CD Stone MB Krause BA. Center-of-pressure parameters used in the assessment of postural control. J Sport Rehabil. (2002) 11:51–66. doi: 10.1123/jsr.11.1.51

[7] Hughes MA Duncan PW Rose DK Chandler JM Studenski SA. The relationship of postural sway to sensorimotor function, functional performance, and disability in the elderly. Arch Phys Med Rehabil. (1996) 77:567–72. 8831473 10.1016/s0003-9993(96)90296-8

[8] Saladin L Voight M. Introduction to the movement system as the foundation for physical therapist practice education and research. Int J Sports Phys Ther. (2017) 12:858–61. doi: 10.26603/ijspt20170858, 29158946 PMC5675361

[9] Cavanaugh JT Kelty-Stephen DG Stergiou N. Multifractality, interactivity, and the adaptive capacity of the human movement system: a perspective for advancing the conceptual basis of neurologic physical therapy. J Neurol Phys Ther. (2017) 41:245–51. doi: 10.1097/NPT.0000000000000199, 28834791 PMC5676558

[10] Bernstein NA. A new method of mirror cyclographie and its application towards the study of labor movements during work on a workbench. Hygiene, Safety Pathol labor. (1930) 5:3–9.

[11] Cofré Lizama LE Panisset MG Peng L Tan Y Kalincik T Galea MP. Postural behaviour in people with multiple sclerosis: a complexity paradox. Gait Posture. (2024) 111:14–21. doi: 10.1016/j.gaitpost.2024.03.013, 38608470

[12] Reynard F Vuadens P Deriaz O Terrier P. Could local dynamic stability serve as an early predictor of falls in patients with moderate neurological gait disorders? A reliability and comparison study in healthy individuals and in patients with paresis of the lower extremities. PLoS One. (2014) 9:e100550. doi: 10.1371/journal.pone.0100550, 24949737 PMC4065053

[13] Muro-De-La-Herran A Garcia-Zapirain B Mendez-Zorrilla A. Gait analysis methods: an overview of wearable and non-wearable systems, highlighting clinical applications. Sensors. (2014) 14:3362–94. doi: 10.3390/s140203362, 24556672 PMC3958266

[14] Cofré Lizama LE Panisset MG Peng L Tan Y Kalincik T Galea MP. Optimal sensor location and direction to accurately classify people with early-stage multiple sclerosis using gait stability. Gait Posture. (2023) 102:39–42. doi: 10.1016/j.gaitpost.2023.02.009, 36889202

[15] Müller R Schreff L Koch L-E Oschmann P Hamacher D. Measuring gait stability in people with multiple sclerosis using different sensor locations and time scales. Sensors. (2021) 21:4001. doi: 10.3390/s21124001, 34200530 PMC8228118

[16] Cofré Lizama LE Strik M Van der Walt A Kilpatrick TJ Kolbe SC Galea MP. Gait stability reflects motor tracts damage at early stages of multiple sclerosis. Mult Scler J. (2022) 28:1773–82. doi: 10.1177/13524585221094464, 35603749

[17] Hilfiker R Vaney C Gattlen B Meichtry A Deriaz O Lugon-Moulin V. Local dynamic stability as a responsive index for the evaluation of rehabilitation effect on fall risk in patients with multiple sclerosis: a longitudinal study. BMC Res Notes. (2013) 6:260. doi: 10.1186/1756-0500-6-260, 23835061 PMC3720262

[18] Comber L Galvin R Coote S. Gait deficits in people with multiple sclerosis: a systematic review and meta-analysis. Gait Posture. (2017) 51:25–35. doi: 10.1016/j.gaitpost.2016.09.026, 27693958

[19] Mehdizadeh S. A robust method to estimate the largest lyapunov exponent of noisy signals: a revision to the rosenstein’s algorithm. J Biomech. (2019) 85:84–91. doi: 10.1016/j.jbiomech.2019.01.013, 30670330

[20] Reynard F Terrier P. Role of visual input in the control of dynamic balance: variability and instability of gait in treadmill walking while blindfolded. Exp Brain Res. (2015) 233:1031–40. doi: 10.1007/s00221-014-4177-5, 25534228

[21] Huisinga JM Yentes JM Filipi ML Stergiou N. Postural control strategy during standing is altered in patients with multiple sclerosis. Neurosci Lett. (2012) 524:124–8. doi: 10.1016/j.neulet.2012.07.020, 22824302

[22] Caronni A Gervasoni E Ferrarin M Anastasi D Brichetto G Confalonieri P. Local dynamic stability of gait in people with early multiple sclerosis and no-to-mild neurological impairment. IEEE Trans Neural Syst Rehabil Eng. (2020) 28:1389–96. doi: 10.1109/TNSRE.2020.2991636, 32356754

[23] Tajali S Mehravar M Negahban H van Dieën JH Shaterzadeh-Yazdi M-J Mofateh R. Impaired local dynamic stability during treadmill walking predicts future falls in patients with multiple sclerosis: a prospective cohort study. Clin Biomech. (2019) 67:197–201. doi: 10.1016/j.clinbiomech.2019.05.013, 31234121

[24] Yentes JM Raffalt PC. Entropy analysis in gait research: methodological considerations and recommendations. Ann Biomed Eng. (2021) 49:979–90. doi: 10.1007/s10439-020-02616-8, 33560467 PMC8051436

[25] Stergiou N. Nonlinear Analysis for human Movement Variability. Florida: CRC Press (2018).

[26] Richman JS Moorman JR. Physiological time-series analysis using approximate entropy and sample entropy. Am J Phys Heart Circ Phys. (2000) 278:H2039–49. doi: 10.1152/ajpheart.2000.278.6.H2039, 10843903

[27] Costa M Goldberger AL Peng C-K. Multiscale entropy analysis of complex physiologic time series. Phys Rev Lett. (2002) 89:068102. doi: 10.1103/PhysRevLett.89.068102, 12190613

[28] Horak FB. Postural orientation and equilibrium: what do we need to know about neural control of balance to prevent falls? Age Ageing. (2006) 35:ii7–ii11. doi: 10.1093/ageing/afl077, 16926210

[29] Busa MA van Emmerik RE. Multiscale entropy: a tool for understanding the complexity of postural control. J Sport Health Sci. (2016) 5:44–51. doi: 10.1016/j.jshs.2016.01.018, 30356502 PMC6188573

[30] Peterka RJ. Sensorimotor integration in human postural control. J Neurophysiol. (2002) 88:1097–1118. doi: 10.1152/jn.2002.88.3.1097, 12205132

[31] Cameron MH Horak FB Herndon RR Bourdette D. Imbalance in multiple sclerosis: a result of slowed spinal somatosensory conduction. Somatosens Mot Res. (2008) 25:113–22. doi: 10.1080/08990220802131127, 18570015 PMC2789668

[32] Kurz MJ Stergiou N. The aging humans neuromuscular system expresses less certainty for selecting joint kinematics during gait. Neurosci Lett. (2003) 348:155–8. doi: 10.1016/S0304-3940(03)00736-5, 12932817

[33] Roeing KL Wajda DA Sosnoff JJ. Time dependent structure of postural sway in individuals with multiple sclerosis. Gait Posture. (2016) 48:19–23. doi: 10.1016/j.gaitpost.2016.04.023

[34] Cattaneo D Jonsdottir J. Sensory impairments in quiet standing in subjects with multiple sclerosis. Mult Scler J. (2009) 15:59–67. doi: 10.1177/1352458508096874, 18845654

[35] Morrison S Rynders C Sosnoff J. Deficits in medio-lateral balance control and the implications for falls in individuals with multiple sclerosis. Gait Posture. (2016) 49:148–54. doi: 10.1016/j.gaitpost.2016.06.036, 27423077

[36] Norbye AD Midgard R Thrane G. Spasticity, gait, and balance in patients with multiple sclerosis: a cross-sectional study. Physiother Res Int. (2020) 25:e1799. doi: 10.1002/pri.1799, 31287210

[37] Cavanaugh JT Guskiewicz KM Stergiou N. A nonlinear dynamic approach for evaluating postural control: new directions for the management of sport-related cerebral concussion. Sports Med. (2005) 35:935–50. doi: 10.2165/00007256-200535110-00002, 16271008

[38] Carpinella I Anastasi D Gervasoni E Di Giovanni R Tacchino A Brichetto G . Balance impairments in people with early-stage multiple sclerosis: boosting the integration of instrumented assessment in clinical practice. Sensors. (2022) 22:9558. doi: 10.3390/s22239558, 36502265 PMC9736931

[39] Cofré Lizama LE He X Kalincik T Galea MP Panisset MG. Sample entropy improves assessment of postural control in early-stage multiple sclerosis. Sensors. (2024) 24:872. doi: 10.3390/s24030872, 38339590 PMC10857195

[40] Fischer OM Missen KJ Tokuno CD Carpenter MG Adkin AL. Postural threat increases sample entropy of postural control. Front Neurol. (2023) 14:1179237. doi: 10.3389/fneur.2023.1179237, 37342783 PMC10277644

[41] Cofré Lizama LE Bastani A van der Walt A Kilpatrick T Khan F Galea MP. Increased ankle muscle coactivation in the early stages of multiple sclerosis. Mult Scler J Exp Transl Clin. (2020) 6:2055217320905870. doi: 10.1177/2055217320905870, 32110431 PMC7016311

[42] Galea MP Cofré Lizama LE Butzkueven H Kilpatrick TJ. Gait and balance deterioration over a 12-month period in multiple sclerosis patients with EDSS scores≤ 3.0. NeuroRehabilitation. (2017) 40:277–84. doi: 10.3233/NRE-161413, 28222549

[43] Strik M Cofré Lizama LE Shanahan CJ Van Der Walt A Boonstra FM Glarin R . Axonal loss in major sensorimotor tracts is associated with impaired motor performance in minimally disabled multiple sclerosis patients. Brain Commun. (2021) 3:fcab032. doi: 10.1093/braincomms/fcab032 34222866 PMC8244644

[44] Chamard Witkowski L Mallet M Bélanger M Marrero A Handrigan G. Cognitive-postural interference in multiple sclerosis. Front Neurol. (2019) 10:913. doi: 10.3389/fneur.2019.00913, 31507517 PMC6716139

[45] Busa MA Jones SL Hamill J van Emmerik RE. Multiscale entropy identifies differences in complexity in postural control in women with multiple sclerosis. Gait Posture. (2016) 45:7–11. doi: 10.1016/j.gaitpost.2015.12.007, 26979875

[46] Etzelmueller MS Yap S-M O’Keeffe C Gaughan M McGuigan C Reilly RB, editors. “Multiscale entropy derived complexity index analysis demonstrates significant mediolateral sway in persons with multiple sclerosis compared to healthy controls.” *2020 42nd Annual International Conference of the IEEE Engineering in Medicine & Biology Society (EMBC)*; (2020): IEEE. 10.1109/EMBC44109.2020.9175672 33019151

[47] Atteya A Elwishy A Kishk N Ismail RS Badawy R. Assessment of postural balance in multiple sclerosis patients. Egypt J Neurol Psychiatr Neurosurg. (2019) 55:7. doi: 10.1186/s41983-018-0049-4

[48] Sun R Hsieh KL Sosnoff JJ. Fall risk prediction in multiple sclerosis using postural sway measures: a machine learning approach. Sci Rep. (2019) 9:16154. doi: 10.1038/s41598-019-52697-2, 31695127 PMC6834625

[49] Santinelli FB Barbieri FA Pinheiro CF Amado AC Sebastião E van Emmerik RE. Postural control complexity and fatigue in minimally affected individuals with multiple sclerosis. J Mot Behav. (2019) 51:551–560. doi: 10.1080/00222895.2019.1567458 30689523

[50] Hunt CM Widener G Allen DD. Variability in postural control with and without balance-based torso-weighting in people with multiple sclerosis and healthy controls. Phys Ther. (2014) 94:1489–98. doi: 10.2522/ptj.20130288, 24903118 PMC4183891

[51] Fino PC. A preliminary study of longitudinal differences in local dynamic stability between recently concussed and healthy athletes during single and dual-task gait. J Biomech. (2016) 49:1983–8. doi: 10.1016/j.jbiomech.2016.05.004, 27207386

[52] Socie MJ Motl RW Pula JH Sandroff BM Sosnoff JJ. Gait variability and disability in multiple sclerosis. Gait Posture. (2013) 38:51–5. doi: 10.1016/j.gaitpost.2012.10.012, 23153835

[53] Sosnoff JJ Sandroff BM Motl RW. Quantifying gait abnormalities in persons with multiple sclerosis with minimal disability. Gait Posture. (2012) 36:154–6. doi: 10.1016/j.gaitpost.2011.11.027, 22424761

[54] Peebles AT Bruetsch AP Lynch SG Huisinga JM. Dynamic balance is related to physiological impairments in persons with multiple sclerosis. Arch Phys Med Rehabil. (2018) 99:2030–7. doi: 10.1016/j.apmr.2017.11.010, 29274726 PMC6014868

[55] van Schooten KS Rispens SM Elders PJ van Dieën JH Pijnappels M. Toward ambulatory balance assessment: estimating variability and stability from short bouts of gait. Gait Posture. (2014) 39:695–9. doi: 10.1016/j.gaitpost.2013.09.020, 24611162

[56] Takakusaki K. Neurophysiology of gait: from the spinal cord to the frontal lobe. Mov Disord. (2013) 28:1483–91. doi: 10.1002/mds.25669, 24132836

[57] Cowan J Dick J Day B Rothwell J Thompson P Marsden C. Abnormalities in central motor pathway conduction in multiple sclerosis. Lancet. (1984) 324:304–7. 10.1016/s0140-6736(84)92683-7 6146860

[58] Hume AL Cant B. Conduction time in central somatosensory pathways in man. Electroencephalogr Clin Neurophysiol. (1978) 45:361–75. 79475 10.1016/0013-4694(78)90188-8

[59] Merton P Morton H Hill D Marsden C. Scope of a technique for electrical stimulation of human brain, spinal cord, and muscle. Lancet. (1982) 320:597–600. 10.1016/s0140-6736(82)90670-5 6125739

[60] Leone C Severijns D Doležalová V Baert I Dalgas U Romberg A. Prevalence of walking-related motor fatigue in persons with multiple sclerosis: decline in walking distance induced by the 6-minute walk test. Neurorehabil Neural Repair. (2016) 30:373–83. doi: 10.1177/1545968315597070, 26216790

[61] Arpan I Fino P Fling B Horak F. Local dynamic stability during long-fatiguing walks in people with multiple sclerosis. Gait Posture. (2020) 76:122–7. doi: 10.1016/j.gaitpost.2019.10.032, 31760315

[62] McLoughlin J Barr C Crotty M Sturnieks D Lord S. Six minutes of walking leads to reduced lower limb strength and increased postural sway in people with multiple sclerosis. NeuroRehabilitation. (2014) 35:503–8. doi: 10.3233/NRE-141143, 25248444

[63] Huisinga JM Mancini M Horak FB. Accelerometry reveals differences in gait variability between patients with multiple sclerosis and healthy controls. Ann Biomed Eng. (2013) 41:1670–9. doi: 10.1007/s10439-012-0697-y, 23161166 PMC3987786

[64] Craig JJ Bruetsch AP Lynch SG Huisinga JM. Altered visual and somatosensory feedback affects gait stability in persons with multiple sclerosis. Hum Mov Sci. (2019) 66:355–62. doi: 10.1016/j.humov.2019.05.018, 31150900 PMC7309345

[65] Gervasoni E Anastasi D Di Giovanni R Solaro C Rovaris M Brichetto G . Uncovering subtle gait deterioration in people with early-stage multiple sclerosis using inertial sensors: a 2-year multicenter longitudinal study. Sensors. (2023) 23:9249. doi: 10.3390/s23229249, 38005634 PMC10674176

[66] Kaipust JP Huisinga JM Filipi M Stergiou N. Gait variability measures reveal differences between multiple sclerosis patients and healthy controls. Mot Control. (2012) 16:229–44. doi: 10.1123/mcj.16.2.229, 22615327

[67] Shema-Shiratzky S Gazit E Sun R Regev K Karni A Sosnoff J . Deterioration of specific aspects of gait during the instrumented 6-min walk test among people with multiple sclerosis. J Neurol. (2019) 266:3022–30. doi: 10.1007/s00415-019-09500-z, 31493037

[68] Pasluosta CF Gassner H Winkler J Klucken J Eskofier BM. An emerging era in the management of Parkinson's disease: wearable technologies and the internet of things. IEEE J Biomed Health Inform. (2015) 19:1873–81. doi: 10.1109/JBHI.2015.2461555, 26241979

[69] Cofré Lizama LE Peng L Kalincik T Galea MP Panisset MG. Multiple sclerosis classification using the local divergence exponent: parameters selection for state-space reconstruction. Sensors. (2025) 25:2819. doi: 10.3390/s25092819, 40363258 PMC12074368

[70] Lin DJ Backus D Chakraborty S Liew S-L Valero-Cuevas FJ Patten C. Transforming modeling in neurorehabilitation: clinical insights for personalized rehabilitation. J Neuroeng Rehabil. (2024) 21:18. doi: 10.1186/s12984-024-01309-w, 38311729 PMC10840185

[71] Lerín-Calvo A Ferrer-Peña R Lerma-Lara S. Advancing neurological rehabilitation: the BRAIN framework for clinical reasoning in Neurophysiotherapy. Brain Sci. (2026) 16:235. doi: 10.3390/brainsci16020235, 41750235 PMC12938968

[72] Abou L Peters J Wong E Akers R Dossou MS Sosnoff JJ. Gait and balance assessments using smartphone applications in Parkinson’s disease: a systematic review. J Med Syst. (2021) 45:87. doi: 10.1007/s10916-021-01760-5, 34392429 PMC8364438

[73] Peters J Abou L Wong E Dossou MS Sosnoff JJ Rice LA. Smartphone-based gait and balance assessment in survivors of stroke: a systematic review. Disabil Rehabil Assist Technol. (2024) 19:177–87. doi: 10.1080/17483107.2022.2072527, 35584288

[74] Abou L Wong E Peters J Dossou MS Sosnoff JJ Rice LA. Smartphone applications to assess gait and postural control in people with multiple sclerosis: a systematic review. Mult Scler Relat Disord. (2021) 51:102943. doi: 10.1016/j.msard.2021.102943, 33873026

[75] Bastani A Panisset MG Cofré Lizama LE. Measuring walking stability with a Mobile phone in older adults: a validation study. Sensors. (2026) 26:2060. doi: 10.3390/s26072060, 41977843 PMC13074950

[76] Panisset MG Galea MP. The effect of COVID-19 lockdowns on exercise and the role of online exercise in Australians with multiple sclerosis. Mult Scler Relat Disord. (2023) 78:104901. doi: 10.1016/j.msard.2023.104901, 37536213

[77] Strongman C Cavallerio F Timmis MA Morrison A. A scoping review of the validity and reliability of smartphone accelerometers when collecting kinematic gait data. Sensors. (2023) 23:8615. doi: 10.3390/s23208615, 37896708 PMC10611257

[78] Carpinella I Bertoni R Anastasi D Cardini R Lencioni T Ferrarin M. Walk longer! Using wearable inertial sensors to uncover which gait aspects should be treated to increase walking endurance in people with multiple sclerosis. Sensors (Basel, Switzerland). (2024) 24:7284. doi: 10.3390/s24227284, 39599061 PMC11598194

[79] Karatsidis A Angelini L Scaramozza M Bartholome E Clinch SP Shen C. Characterizing gait in people with multiple sclerosis using digital data from smartphone sensors: a proposed framework. Mult Scler J. (2025) 31:512–28. doi: 10.1177/13524585251316242, 39963834 PMC12008473

[80] Klotzbier TJ Korbus H Johnen B Schott N. Evaluation of the instrumented timed up and go test as a tool to measure exercise intervention effects in nursing home residents: results from a PROCARE substudy. Ger J Exerc Sport Res. (2021) 51:430–42. doi: 10.1007/s12662-021-00764-0

